# Repeated cold stress, an animal model for fibromyalgia, elicits proprioceptor-induced chronic pain with microglial activation in mice

**DOI:** 10.1186/s12974-024-03018-6

**Published:** 2024-01-18

**Authors:** Koji Wakatsuki, Sumiko Kiryu-Seo, Masaya Yasui, Hiroki Yokota, Haruku Kida, Hiroyuki Konishi, Hiroshi Kiyama

**Affiliations:** 1 Department of Functional Anatomy and Neuroscience, Nagoya University Graduate School of Medicine, 65 Tsurumaicho, Showa-Ku, Nagoya, Aichi 466-8550 Japan; 2 Department of Judo Seifuku and Health Sciences, Tokoha University, 1230 Miyakoda-Cho, Kita-Ku, Hamamatsu, Shizuoka 431-2102 Japan; 3 Department of Mechanical Engineering, Meijo University, 1-501 Shiogamaguchi, Tenpaku-Ku, Nagoya, Aichi 468-0073 Japan

**Keywords:** Fibromyalgia, Myalgic encephalomyelitis, Intermittent cold stress, Chronic fatigue syndrome, Neuronal inflammation, Pain, Microglia

## Abstract

**Background:**

Fibromyalgia is characterized by chronic pain, fatigue, and other somatic symptoms. We have recently revealed that proprioceptor hyperactivation induces chronic pain in a rat model of myalgic encephalomyelitis. The present study explores whether similar proprioceptor-induced pain is elicited in a mouse model of fibromyalgia.

**Methods:**

Repeated cold stress (RCS) was used as a fibromyalgia model. Pain behavior was examined using the von Frey test, and neuronal activation was examined immunohistochemically as activating transcription factor (ATF)3 expression. The *Atf3*:BAC transgenic mouse, in which mitochondria in hyperactivated neurons are specifically labeled by green fluorescent protein, was used to trace the activated neuronal circuit. PLX3397 (pexidartinib) was used for microglial suppression.

**Results:**

RCS elicited long-lasting pain in mice. ATF3, a marker of cellular hyperactivity and injury, was expressed in the lumbar dorsal root ganglion (DRG) 2 days after RCS initiation; the majority of ATF3-expressing DRG neurons were tropomyosin receptor kinase C- and/or vesicular glutamate transporter 1-positive proprioceptors. Microglial activation and increased numbers of microglia were observed in the medial part of the nucleus proprius 5 days after RCS initiation, and in the dorsal region of the ventral horn 7 days after RCS. In the ventral horn, only a subset of motor neurons was positive for ATF3; these neurons were surrounded by activated microglia. A retrograde tracer study revealed that ATF3-positive motor neurons projected to the intrinsic muscles of the foot (IMF). Using *Atf3*:BAC transgenic mice, we traced hyperactivated neuronal circuits along the reflex arc. Green fluorescent protein labeling was observed in proprioceptive DRG neurons and their processes originating from the IMF, as well as in motor neurons projecting to the IMF. Microglial activation was observed along this reflex arc, and PLX3397-induced microglial ablation significantly suppressed pain behavior.

**Conclusion:**

Proprioceptor hyperactivation leads to local microglial activation along the reflex arc; this prolonged microglial activation may be responsible for chronic pain in the present model. Proprioceptor-induced microglial activation might be the common cause of chronic pain in both the fibromyalgia and myalgic encephalomyelitis models, although the experimental models are different.

**Supplementary Information:**

The online version contains supplementary material available at 10.1186/s12974-024-03018-6.

## Background

Functional somatic syndrome (FSS) is a term that is applied to several related syndromes that are characterized more by symptoms, suffering, and disability than by consistently demonstrable tissue abnormalities [1]. FSSs include chronic fatigue syndrome (CFS)/myalgic encephalomyelitis (ME), fibromyalgia, irritable bowel syndrome, multiple chemical sensitivity, sick building syndrome, repetitive stress injury, and Gulf War syndrome. These disorders have many shared symptoms; chronic pain, fatigue, and sleep disorders are particularly common. Wessely et al. [2] reviewed FSSs and concluded that overlaps exist between the individual syndromes, and that the similarities between the syndromes outweigh the differences.

Among FSSs, ME/CFS is a disorder characterized by overwhelming or severe fatigue accompanied by pain, sleep disorder, depression and cognitive disorders [3], whereas fibromyalgia is a disorder characterized by widespread musculoskeletal pain accompanied by severe fatigue, sleep disorder, anxiety and depression [4]. CFS/ME and fibromyalgia, thus, have very similar symptoms, such as chronic pain and severe fatigue, although the mechanisms remain unknown. We have previously attempted to explore the molecular mechanism underlying CFS/ME using a continuous stress-loading rat model that partially mimics the symptoms associated with CFS/ME [5–12]. In this model, rats were housed in a cage with a thin level of water (1.5 cm in depth) for 5 days; multiple stresses including sleep disturbances and psychological stress were presumed to elicit severe fatigue and chronic pain in the animal. Notably, we found that proprioceptor hyperactivation further activated microglia in the dorsomedial part of the spinal cord, and that the suppression of microglial activity reduced pain. Together, these findings suggest that microglia may be a key player in the initiation and maintenance of abnormal pain in patients with CFS [13]. We thus expect that a similar mechanism may elicit chronic pain in fibromyalgia. In the present study, we therefore used a mouse model of fibromyalgia to explore the molecular mechanisms underlying chronic pain in this model.

Repeated (intermittent) cold stress (RCS or ICS) was originally known as specific alteration of rhythm in temperature stress [14]. In RCS, the environmental temperature is rapidly changed at short intervals (e.g., 30 min) between normal and cold temperatures. It is one of the optimal rodent models of fibromyalgia. Rats were originally used in this model, and were reported to exhibit persistent pain-related behaviors [15]. Montserrat-de la Paz et al. [16] evaluated whether this model was also suitable for creating a mouse model of fibromyalgia, and demonstrated that ICS (i.e., RCS) induced mechanical allodynia, thermal allodynia, and hyperalgesia as well as behavioral changes related to cognitive disturbances, anxiety, and depression. The authors thus concluded that ICS is a useful animal model for assessing the underlying mechanisms involved in fibromyalgia development. Nishiyori and Ueda [17] also reported that this mouse model is a suitable fibromyalgia model. Mice showed bilateral hindlimb allodynia lasting more than 12 days as well as thermal hyperalgesia lasting 15 days. Intriguingly, mice exposed to constant (rather than intermittent) cold stress showed only transient allodynia, and systemic gabapentin treatment had complete anti-allodynic effects in this model. Together, these findings support the validity of RCS as a fibromyalgia model. We thus used an RCS model in the present investigation to explore the central causative mechanism of fibromyalgia. Furthermore, to analyze the neuronal circuit that is activated by RCS, we used activating transcription factor 3 (*Atf3*):bacterial artificial chromosome (BAC) transgenic (Tg) mice to label mitochondria with green fluorescent protein (GFP) in a hyperactivation- or damage-induced manner, under the control of a regulatory element of *Atf3* [18–21]. The use of this Tg mouse allows the tracing of activated neuronal circuits in the spinal cord. Our findings suggest that the mechanisms of chronic pain in CFS/ME and fibromyalgia are common; proprioceptor hyperactivation induced microglial activation, and microglial activation caused chronic pain.

## Methods

### Animals

All wild-type C57BL/6NCrSIc mice (SLC Corporation, Shizuoka, Japan) used in the present experiments were male (8–13 weeks old).

The *Atf3*:BAC Tg mice were originally generated according to general BAC modification protocols; the precise procedures are described by Kiryu-Seo et al. [18, 20]. The *Atf3*:BAC Tg mice were crossed with C57BL/6Ncr mice for at least 10 generations. Hemizygous mice were fertile and viable without apparent neurological abnormalities. Tail lysates from the mice were used for genotyping with specific primer sets: *Atf3*:BAC Tg-F 5′-CAATAAGATGGAGTACAACTACAACGC-3′ and *Atf3*:BAC Tg-R 5′-GACTCTTTCCACAACTATCCAACTCAC-3′. All *Atf3*:BAC Tg mice used in the present experiments were male (9–18 weeks old).

Mice were kept in a temperature-controlled room (23 °C ± 1 °C) with a 12-h light/dark cycle (lights on from 9:00 am to 9:00 pm), with free access to food and water. The animals were reared according to the Guide for the Care and Use of Laboratory Animals (National Research Council, 1996). The present study was conducted in accordance with the Nagoya University Animal Experimentation Regulations (permission no. M220103-002 and M230157-002), the Basic Guidelines for the Proper Conduct of Animal Experiments in Japanese University Research Institutions, and the Ethical Guidelines of the International Association for the Study of Pain [22].

### RCS-induced fibromyalgia model

**Fig. 1:**
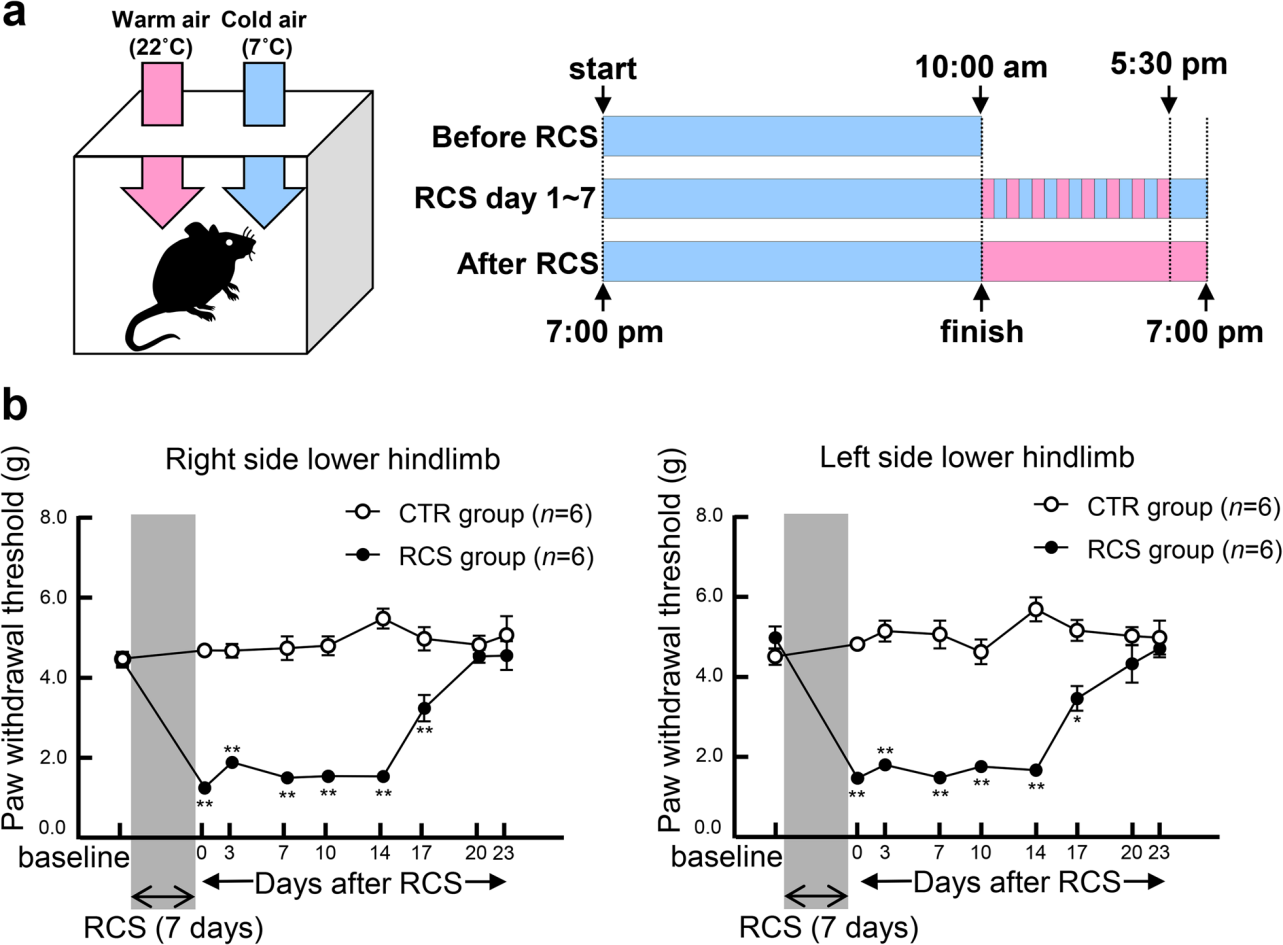
Experimental protocol for RCS and assessing pain behavior. **a** Schematic illustration of the RCS protocol. Mice were first kept at 7 °C in the RCS chamber from 7:00 pm to 10:00 pm (Before RCS). The mice were subjected to temperature changes between 7 °C and 22 °C every 30 min from 10:00 am to 5:30 pm, and were then maintained at 7 °C from 5:30 pm to 10:00 am for 7 days (RCS). After 7 days of RCS exposure, the RCS schedule ended at 10:00 am (After RCS). **b** Hind PWT was measured in the left and right hindlimbs using the electronic von Frey test. The PWT of the RCS group remained significantly lower than that of the untreated control (CTR) group in both the left and right hindlimbs until day 17 after RCS. *n* = 6 mice for each group. *p < 0.001, **p < 0.0001. Error bars indicate the SEM. Two-way ANOVA with Bonferroni's multiple comparisons test

To create an animal model of fibromyalgia, mice were exposed to RCS using an automated RCS chamber (made by the Yokota Laboratory, Meijo University). Mice were kept in cages with metal mesh floors (width × depth × height: 93 × 205 × 127 mm; TM-PC-S, Tokiwa Inc., Tokyo, Japan). RCS was performed in a chamber containing six of these cages, and cold and warm air flowed in alternately every 30 min from 10:00 am to 5:30 pm, thus changing the temperature in the cages from room temperature (22 °C) to cold temperature (7 °C). The lower temperature setting (7 °C) was determined by examining skin tissue damage, inflammation, and edema; a temperature of 7 °C did not lead to any apparent damage to the skin. Inside the automated RCS chamber, light-emitting diode lights were automatically turned on during the daytime (between 8:00 am and 8:00 pm) to minimize any effects on the sleep cycles of the mice. The precise RCS protocol is shown in Fig. 1a. First, the cage containing the mice was placed in a 7 °C chamber at 7:00 pm on the day before the start of RCS. The next day was the first day of RCS, with alternating exposure to cold and room temperatures for 30 min each from 10:00 am to 5:30 pm. After 7 days of RCS exposure, mice were removed from the chamber at 10:00 am and transferred to their normal cages in a clean room.

Female mice altered the pain behavior and microglial activation in response to RCS (Additional file 2: Fig. S1). Previous papers used both male and female mice without any difference in RCS model [17, 31]. In this study, we used male mice to avoid the disturbance by estrous cycle in female mice [23].

### Pain behavioral test

Pain behavior in RCS-exposed mice was examined using the electronic von Frey test on the plantar surface of the hind paw. An electronic von Frey esthesiometer (No. 2391; IITC, Los Angeles, CA, USA) was used for the quantitative measurement of the mechanical hind paw withdrawal threshold (PWT). A blunt attachment (tip diameter: 1 mm) was fabricated in our laboratory. The chip was slowly applied to the center of the plantar area of the hind paw and pressed gradually; the pressure was automatically measured when the mouse spontaneously lifted its hind paw. Five trials were performed per leg, and the PWT was obtained by removing the minimum and maximum values and calculating the average of the remaining three values. The left and right legs were alternately stimulated, with an interval of at least 30 s between each stimulus and the next.

### Immunofluorescence staining

Mice were perfused transcardially with Zamboni solution (2% paraformaldehyde and 0.2% picric acid in 0.2 M phosphate buffer). After perfusion, the spinal cord with the DRG was removed, postfixed, immersed in 30% sucrose in 0.1 M phosphate-buffered saline (PBS) for cryoprotection, and stored at 4 °C. For the muscles, the entire hindlimb was treated using the same procedure as for the spinal cord, and the examined muscles were removed. The obtained specimens were then embedded in Tissue-Tek^®^ Optimal Cutting Temperature Compound (Sakura Finetek Japan K.K., Tokyo, Japan) and frozen in liquid nitrogen. Serial slices were prepared using a cryostat and attached to the slide glasses. The DRG, spinal cord, and muscle specimens were sliced into 10-, 20-, and 40-μm-thick sections, respectively. Sections were blocked with 1% bovine serum albumin and 0.3% Triton-X100 in 0.1 M PBS, and were then incubated with the appropriate primary antibody overnight. Subsequently, the sections were incubated with secondary antibody for 90 min. The primary and secondary antibodies used for immunofluorescence staining are listed in Additional file 1: Table S1.

For the whole-mount observation of nerves, the dorsal and ventral roots were carefully removed, washed in PBS, and sealed with mounting medium before being observed under a microscope. Images were acquired using a confocal laser microscope (TiE-A1R, × 10, × 20 and × 40 objectives or × 60 water immersion objective, Nikon Corporation, Tokyo, Japan) or a fluorescence microscope (BZ-X800, × 10, × 20, and × 40 objectives, Keyence Corporation, Osaka, Japan; BX53, × 10 and × 40 objectives, Olympus Corporation, Tokyo, Japan).

### Retrograde neuronal tracing

To identify the muscles that were innervated by GFP-positive motor neurons in the spinal cord of *Atf3*:BAC Tg mice, cholera toxin B subunit (CTXb) conjugated with Alexa 647 (Fluorochrome, Denver, CO, USA) was injected into various muscles in the hindlimb of Tg mice after RCS exposure and traced to motor neurons in the spinal cord. For each target muscle, 2 µL of CTXb-containing solution (1 mg/mL) was injected. After the injection, animals were kept normally for 5 days, followed by fixation and immunostaining.

### Quantitative reverse transcription polymerase chain reaction (qRT-PCR)

Hind paw skin (hairless sole) and intrinsic muscles of the foot (IMF) tissue were collected within 24 h after RCS completion; they were immediately frozen in liquid nitrogen and stored at − 80 °C. Messenger RNA expression levels of interleukin (IL)-1β, IL-6, and tumor necrosis factor-α (TNF-α) were quantified in the muscles and skin of RCS-exposed mice. Total RNA was extracted using an RNeasy^®^ Plus Mini Kit (Qiagen, Hilden, Germany) and quantified using a spectrophotometer (NanoDrop 2000, Thermo Fisher Scientific, Waltham, MA, USA). Reverse transcription was performed using SuperScript III (Invitrogen, Waltham, MA, USA) to synthesize cDNA from total RNA. Finally, qRT-PCR was performed on a StepOnePlus (Applied Biosystems, Waltham, MA, USA) using Fast SYBR Green Master Mix (Applied Biosystems).

The primers were as follows: TNF-α forward: 5′-AGCCGATGGGTTGTACCTTGTCTA-3′ and reverse: 5′-TGAGATAGCAAATCGGCTGACGGT-3′; IL-1β forward: 5′-ACAGAATATCAACCAACAAGTGATATTCTC-3′ and reverse: 5′-GATTCTTTCCTTTGAGGCCCA-3′; IL-6 forward: 5′-ATCCAGTTGCCTTCTTGGGACTGA-3′ and reverse: 5′-TAAGCCTCCGACTTGTGAAGTGGT-3′; and glyceraldehyde-3-phosphate dehydrogenase (GAPDH) forward: 5′-CAAGGTCATCCCAGAGCTGA-3′ and reverse: 5′-CGGCACGTCAGATCCACGAC-3′.

The conditions for high-speed qRT-PCR were one cycle of 95 °C × 20 s, 40 cycles of 95 °C × 3 s, and 1 cycle of 60 °C × 30 s. The specificity of amplicons was confirmed by melt curve analysis. Data were normalized using GAPDH, and fold changes were determined using the 2^−ΔΔCt^ method.

### Blood tests

On the day of RCS termination, blood was collected from the mice and centrifuged (10,000×*g* for 5 min); 100 μL of the serum sample was diluted by saline (Otsuka Pharmaceutical Factory, Tokyo, Japan). The diluted blood sample was used for analysis of C-reactive protein and creatine kinase (SRL International, Inc., Tokyo, Japan).

### PLX3397 treatment

PLX3397 (pexidartinib; ChemScene LLC., Monmouth Junction, NJ, USA), a known inhibitor of colony-stimulating factor-1 receptor, was suspended at 25 mg in 400 μL of 0.5% methyl cellulose solution (Fujifilm, Tokyo, Japan) and administered orally to wild-type mice for 3 days at 6:00 am and 6:00 pm [24], with RCS. The same schedule was then followed during the RCS exposure period.

### Histological quantification

All tissue sections were statistically processed by randomly selecting at least two sections per mouse. In the images acquired with a microscope (Olympus BX53, Keyence BZ-X800), each DRG neuron was identified as a dot and the numbers were counted using the image processing software GIMP 2.10.30 (GIMP Development Team) and the image processing and analysis software ImageJ (National Institutes of Health, Bethesda, MD, USA). Similarly, lumbar spinal cord sections from RCS mice were randomly selected, and a 20,000 μm^2^ field of observation was cut from the images acquired by fluorescence microscopy using GIMP. Next, ImageJ was used to quantify the numbers of microglia (cells that co-expressed Iba1 and 4′,6-diamidino-2-phenylindole; *n* = 5 or 6 mice per group). The area occupied by GFP-positive fibers in the spinal cord was also quantified in the same manner (*n* = 5 mice per group). ChAT-positive and GFP- and ATF3-positive neurons in the spinal cord were similarly quantified (*n* = 4 or 5 mice per group). Images were adjusted identically and appropriately in each group, and were examined using the editing software supplied with the microscope (TiE-A1R Nikon: NIS-Elements Analysis; Keyence BZ-X800: BZ-X800 Analyzer) or GIMP.

### Statistical analyses

All data are presented as the mean ± standard error of the mean (SEM). Statistical analysis was performed using Prism 9.3.1(471) (GraphPad Software, La Jolla, CA, USA); the significance level was set at *p* < 0.05. Data for mechanical withdrawal thresholds over time were analyzed using two-way analysis of variance (ANOVA) and Bonferroni’s multiple comparisons test. Data examining changes in microglia over time in wild-type mice, and in ATF3-positive cells in the DRG, were analyzed using one-way ANOVA and Dunnett's multiple comparisons test. The classification of GFP-positive cells in the DRG of Tg mice was analyzed using one-way ANOVA with Bonferroni’s multiple comparisons test. The unpaired t-test was used to analyze ATF3- or GFP-positive cells in the DRG of Tg mice, ATF3- or GFP-positive motor neurons in the spinal cord of Tg mice, microglia and GFP expression by site in the spinal cord of Tg mice, as well as changes in microglia after PLX3397 administration. Data for mechanical withdrawal thresholds after PLX3397 administration were analyzed using paired *t*-test.

## Results

### RCS loading elicits long-lasting pain in mice

The paw withdrawal threshold (PWT) of the lower hindlimb was measured to examine nociceptive sensitivities. In control group without RCS, PWT was not altered during the observation period. In RCS group, PWT was reduced to 1.44 g in left side (baseline 4.94 g) and 1.24 g in right side right (baseline 4.42 g) immediately after RCS loading (RCS stimuli for 7 days) (Fig. 1a). RCS group decreased the threshold with statistically significance, compared with control group (*p* < 0.0001) and remained it for 17 days after RCS, suggesting chronic hyperalgesia. The decrease in PWT recovered to control levels by 20 days after RCS loading (Fig. 1b).

### Microglial activation and accumulation in the spinal cord

**Fig. 2:**
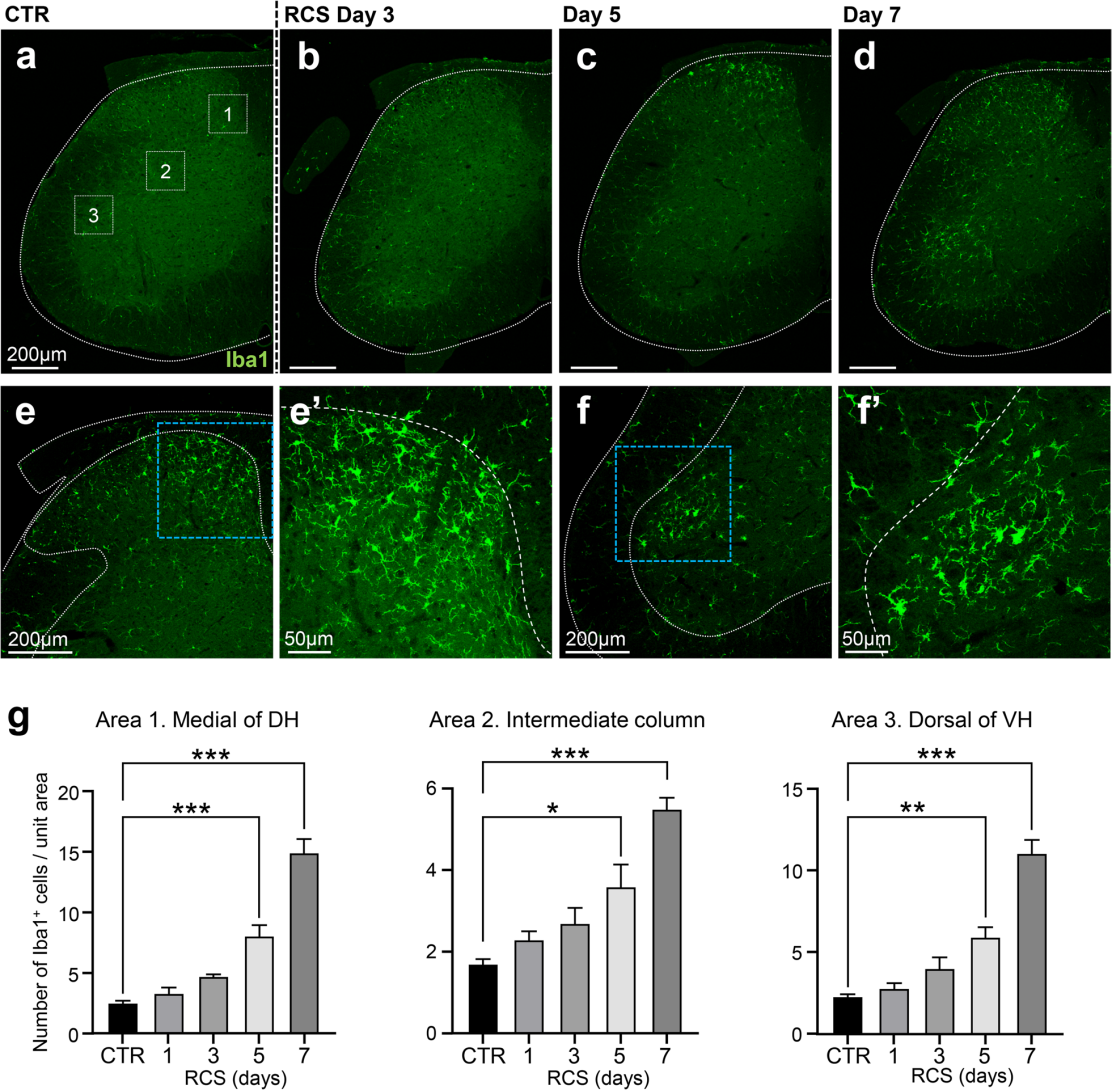
Accumulation of activated microglia in the lumbar spinal cord during RCS loading. **a–d** The appearance and accumulation of activated microglia in the L5 spinal cord along the RCS protocol. Note that a significant accumulation of microglia (Iba1-positive cells) was observed in the medial part of the DH from day 5 of RCS. A slight accumulation of microglia was observed in the VH from day 5 of RCS; this was markedly increased at day 7. The dotted areas, designated 1, 2, and 3 in **a**, are the areas used for quantification in **g**. **e** Higher-magnification images of Iba1-positive microglia in the DH of the L5 spinal cord after 7 days of RCS loading (day 7). The area surrounded by a blue dotted rectangle is magnified in **e’**. **f** Magnified images of activated microglia in the VH of the lumbar spinal cord on day 7 of RCS loading. The area surrounded by a blue dotted rectangle is magnified in **f’**. Note that both **e’** and **f’** clearly demonstrate that the accumulated microglia have an activated shape. **g** Quantification of the numbers of microglia in Area 1: medial area of the DH; Area 2: intermediate column; and Area 3: dorsal area of the VH; surrounded by dotted squares in the image in **a**. *n* = 5 mice for each group. *p < 0.01, **p < 0.001, ***p < 0.0001. Error bars indicate the SEM. One-way ANOVA with Dunnett's multiple comparisons test. The white dotted lines indicate the edge of spinal cord in **a**–**d** and the edge of spinal cord and gray matter in **e–f’**. Scale bars = 200 μm in **e**, **f**, and 50 μm in **e’, f’**

Accumulations of Iba1-positive microglia were observed in both the dorsal horn (DH) and ventral horn (VH) of the lumbar spinal cord during and after RCS (Fig. 2a–d). In the control mice and at 3 days of RCS, no obvious microglial accumulation was observed in any region of the spinal cord, although sparse microglia were localized evenly throughout this region. However, after 5 days of RCS loading (Fig. 2c), significant microglial accumulation was observed in the medial part of the DH (area 1 in Fig. 2a); microglial numbers were further increased after 7 days of RCS (Fig. 2d). In the VH, microglial accumulation began after 5 days of RCS in the dorsal part of the VH (area 3 in Fig. 2a), specifically; microglial numbers were markedly increased after 7 days of RCS (Fig. 2d). When the microglia were observed under higher-power magnification, all of the accumulated microglia had relatively thick and short processes, suggesting an activated state (Fig. 2e–f’). Concomitantly, a slight accumulation of microglia was observed in the intermediate part of the spinal cord (area 2 in Fig. 2a); however, the increase in this area was relatively small compared with that of other regions (areas 1 and 3) (Fig. 2g). The increased number of microglia was returned to a normal level when the pain behavior became to normal level. The present data suggest that a longer RCS period leads to greater numbers of microglia in some areas of the spinal cord.

Although microglial activation and proliferation were observed in the RCS model, no significant peripheral inflammation signatures, such as increases in C-reactive protein and creatine kinase, were identified in the peripheral blood of RCS model mice (Additional file 3: Fig. S2a). Furthermore, little changes in the mRNA expression of IL-1β, IL-6, or TNF-α were observed in peripheral areas, such as the plantar skin and muscle (Additional file 3: Fig. 2b, c).

### RCS elicits ATF3 expression in subsets of DRG and motor neurons

**Fig. 3:**
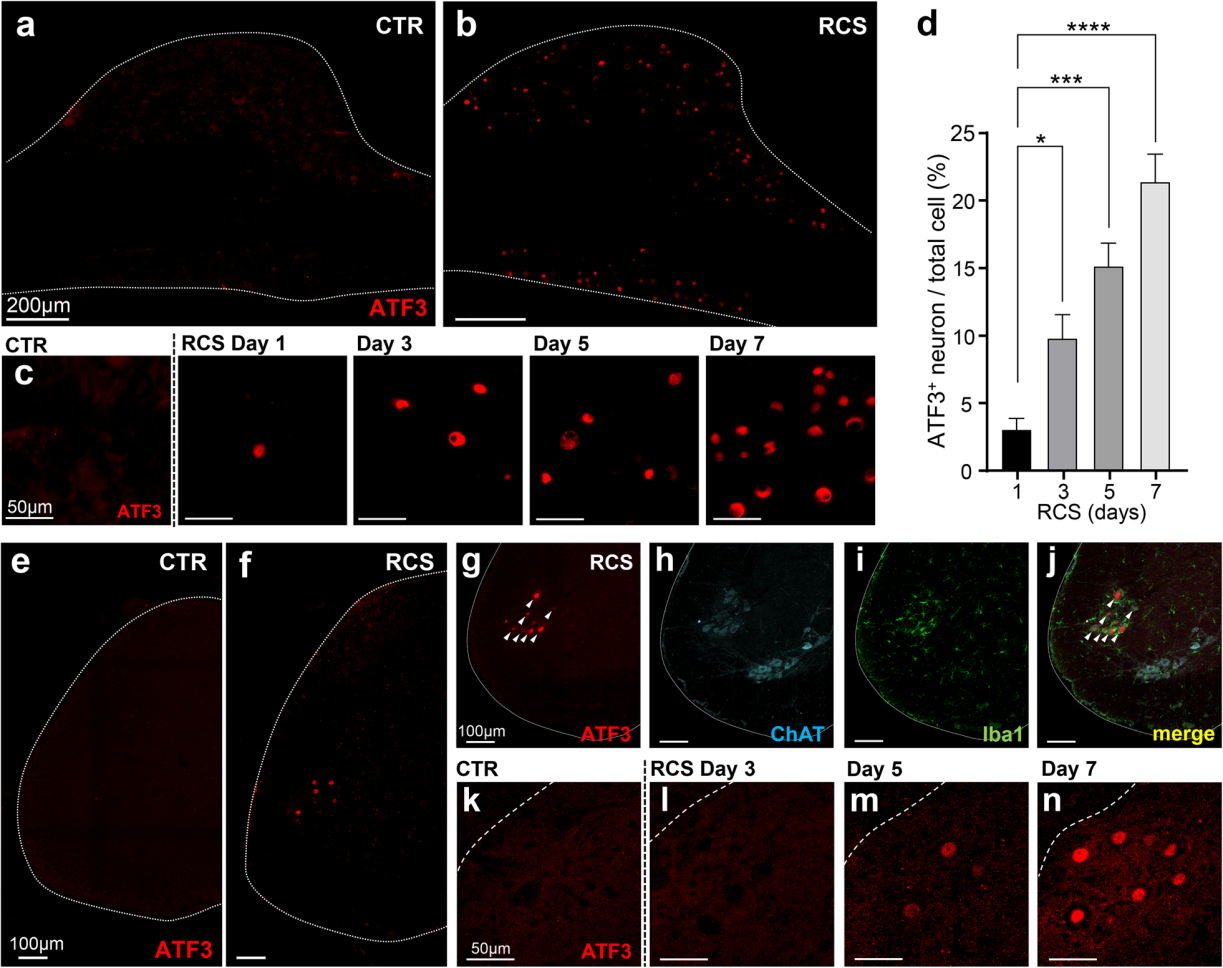
ATF3 expression in DRG and VH neurons. **a, b** ATF3 expression in DRG neurons of control (CTR) and RCS mice (7 days of RCS). The white dotted lines indicate the edge of the DRG. **c** Time-dependent expression of ATF3 in DRG neurons after RCS loading. **d** Quantification of the number of ATF3-positive DRG neurons after RCS. Note that a small number of ATF3-positive cells was observed from day 1; this increased in a time-dependent manner. *n* = 5 mice for each group. *p < 0.05, ***p < 0.001, ****p < 0.0001. Error bars indicate the SEM. One-way ANOVA with Dunnett’s multiple comparisons test. **e, f** ATF3 expression in spinal cord of control (CTR) and RCS mice (7 days of RCS). **g–j** Immunostaining of ATF3, ChAT and Iba1 in spinal cord at 7 days of RCS. ATF3-positive neurons (arrowheads) were ChAT-positive in the dorsal part of the VH and surrounded by Iba1-positive microglia after RCS loading. The white dotted lines show the edge of spinal cord. **k–n** Time-dependent expression of ATF3 in the VH of spinal code after RCS. The white dotted lines in **k–n** show the edge of gray matter in spinal cord. Scale bars, 200 μm in **a**, **b**, 50 μm in **c**, **k**–**n** and, 100 μm in **e–j**

ATF3 is a transcription factor that is present at less than the detection level in neuronal tissues; it is expressed specifically in injured neurons. Recently, we found that the expression of ATF3 is induced not only in axon injury but also in stress-induced hyperactivation caused by disease such as ALS [20]. In line with this, we used ATF3 as an indicator for an RCS-induced hyperactivation. Marked expression of ATF3 was observed in a group of neurons in the DRG during RCS (Fig. 3a, b). The number of ATF3-positive neurons in L5 exceeded those in L4 and L6; we therefore used the L5 DRG for further quantification studies. The expression of ATF3 in the DRG was observed from day 1 of RCS loading, when only a few positive cells were present per section; however, very high expression was observed at day 7 of RCS (Fig. 3c, d). The ATF3-positive cell rate (as a percentage of the total cells per section) was 3.2% on day 1 of RCS, 9.9% on day 3, 15.2% on day 5, and 21.5% on day 7 (Fig. 3d), suggesting that ATF3 expression increases in a stress period-dependent manner.

A subset of neurons located in the dorsal part of the VH also expressed ATF3, although this expression was not observed in the control spinal cord (Fig. 3e–j). We confirmed that ATF3-positive cells expressed choline acetyltransferase (ChAT), which is a marker for motor neurons (Fig. 3g–j). Notably, most of the ATF3-positive neurons were surrounded by activated microglia. ATF3 expression first appeared on RCS loading day 5 and was increased in number on day 7 of RCS loading, suggesting that ATF3 expression in the VH occurs later than that in the DRG (Fig. 3k–n).

### Expression of ATF3 and GFP in *Atf3*:BAC Tg mice in response to RCS

**Fig. 4:**
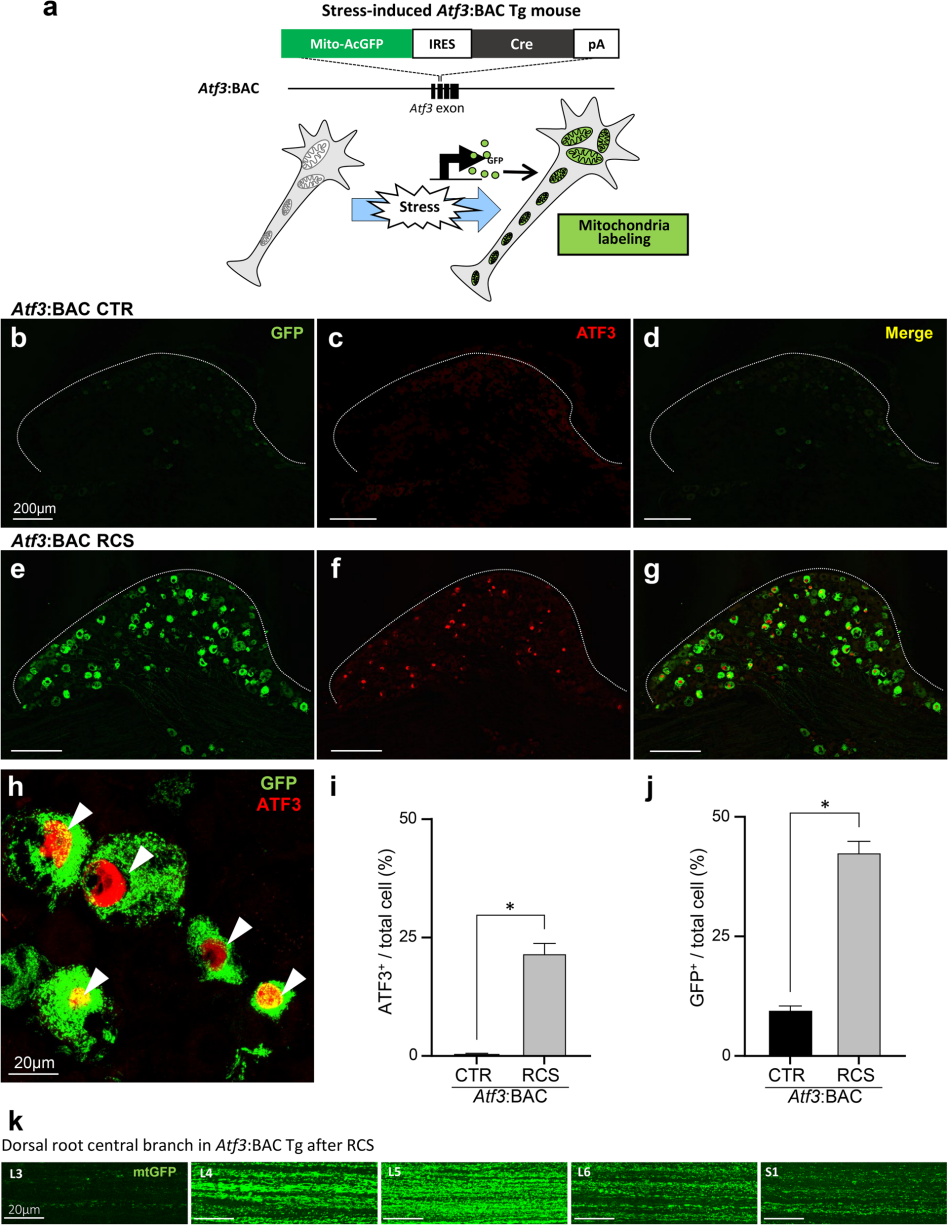
ATF3-positive neurons are labeled by GFP in* Atf3*:BAC Tg mice after RCS loading. **a** *Atf3*:BAC Tg mouse labels mitochondria with GFP upon the expression of ATF3. **b–g** GFP and ATF3 expression in DRG neurons of control (CTR) (**b–d**) and RCS (**e–g**) mice at 7 days of RCS loading. The white dotted lines indicate the edge of the DRG. **h** High-power magnification photographs clearly demonstrate that cell nuclei were stained by ATF3 (red) (arrowheads), whereas the cytoplasm was labeled with GFP (green). **i, j** Percentages of ATF3- (**i**) or GFP-positive (**j**) neurons, as a percentage of DAPI-positive DRG neurons. *n* = 5 mice, *p < 0.0001. Error bars indicate the SEM. Unpaired *t*-test. **k** Whole-mount images showing that GFP-tagged mitochondria (mtGFP) were present in the central branch of the dorsal root from L3 to S1. Scale bars, 200 μm in **b–g**, 20 μm in **h, k**

We next used *Atf3*:BAC Tg mice to further analyze the characteristics of RCS-responsive neurons. As shown in Fig. 4a, the *Atf3*:BAC Tg mouse is designed to express GFP with a mitochondria-targeting signal and *Cre*-recombinase under the transcriptional regulation of *Atf3* [20]. Thus, only mitochondria in hyperactivated or injured neurons are labeled with GFP.

We first compared the expression of GFP and ATF3 in the DRG before and after RCS. As expected, most GFP-positive cells also expressed ATF3 (Fig. 4b–g). As shown in Fig. 4h, ATF3 was localized in the nucleus, whereas GFP staining was observed in cytoplasmic regions. In DRG sections before RCS, 0.4% of total DRG neurons expressed ATF3, and 9.7% of neurons expressed GFP. In contrast, after RCS, 21.7% of DRG neurons expressed ATF3, and 42.6% of neurons expressed GFP (Fig. 4i, j). 82.9% of ATF3-expressing cells expressed GFP after RCS. One possible explanation for the finding that ATF3 and GFP did not perfectly coincide may be the loss of nuclei through the sectioning and staining processes. *Atf3*:BAC Tg mouse visualized the central branch of the dorsal root from L3 to S1 with GFP. The most abundant levels of GFP-positive fibers were observed in the L5 root; the numbers of positive fibers became fewer in the roots next to L5 (Fig. 4k).

**Fig. 5:**
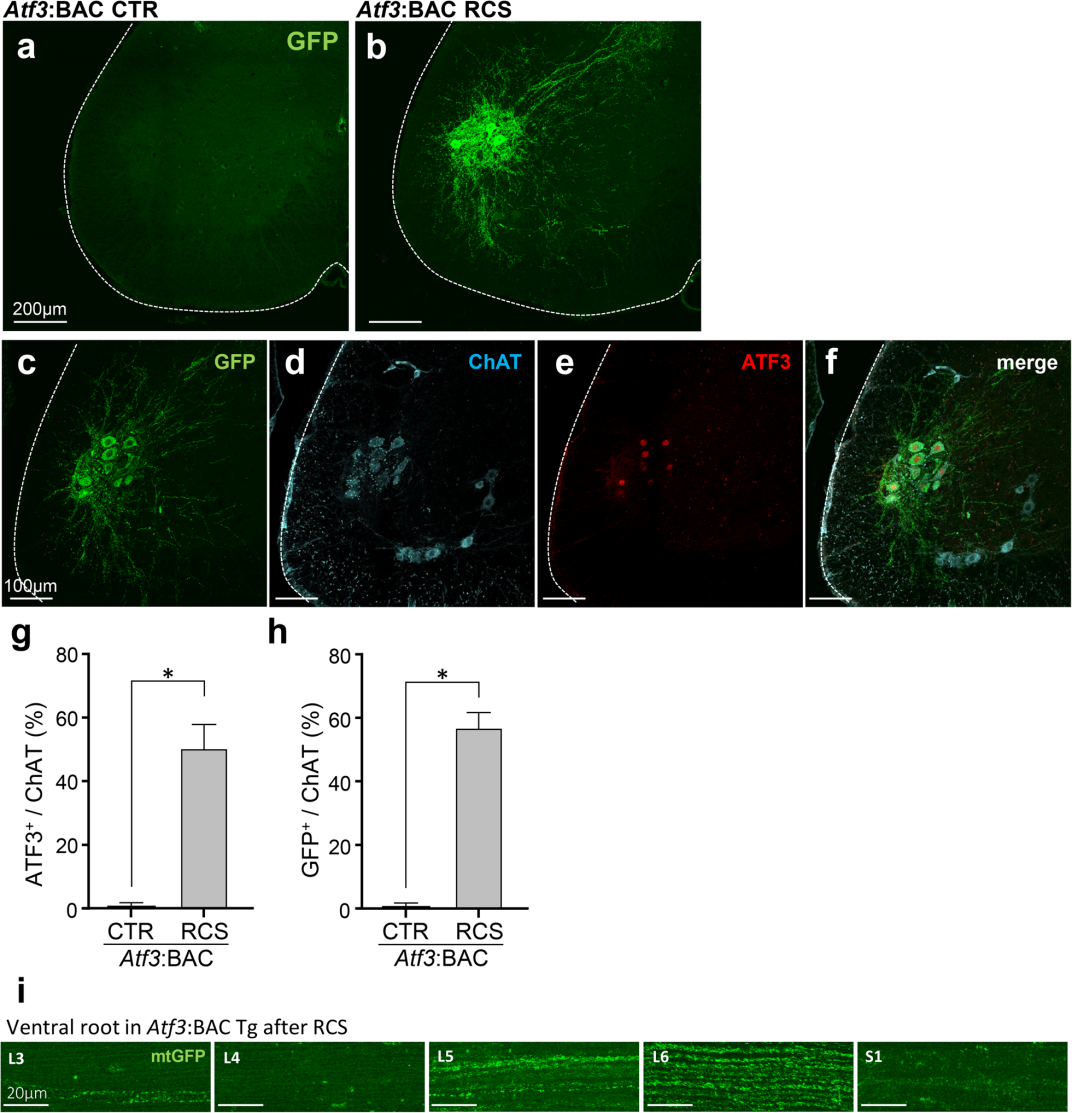
GFP-positive neurons in the VH of* Atf3*:BAC Tg mice after RCS are motor neurons. **a**, **b** GFP expression in the VH of the spinal cord of *Atf3*:BAC Tg mouse before (*Atf3*:BAC CTR) **(a)** and after 7 days of RCS (*Atf3*:BAC RCS) (**b**). **c–f** GFP expression (green) in the dorsal region of VH of the spinal cord in ATF3- (red) and ChAT-positive (light blue) motor neurons. **g**, **h** Graph showing the quantification of ATF3- or GFP-positive neurons, as a percentage of ChAT-positive neurons. *n* = 4 mice for CTR, *n* = 5 mice for RCS group. *p < 0.0001. Error bars indicate the SEM. Unpaired *t*-test. **i** Whole-mount images showing that GFP-tagged mitochondria (mtGFP) were present in the ventral root from L3 to S1. The white dotted lines indicate the edge of spinal cord in **a–f**. Scale bars, 200 μm in **a, b**, 100 μm in **c–f** and 20 μm in **i**

In the VH, almost all GFP-positive neurons expressed ATF3 in *Atf3*:BAC Tg mouse after RCS (Fig. 5a, b). To determine the GFP-/ATF3-positive cell types, we double-labeled sections using ChAT antibody. Almost all GFP-positive cells also expressed ChAT, suggesting that they are motor neurons. Notably, GFP-/ATF3-positive motor neurons were restricted to the dorsal area of the VH. GFP and ATF3 were not expressed in motor neurons in the ventral region of the VH (Fig. 5c–f). Comparing all ChAT-positive motor neurons in sections of the lumbar spinal cord, before RCS, 0.9% of motor neurons expressed ATF3, and 0.9% of neurons expressed GFP. In contrast, after RCS, 50.5% of motor neurons expressed ATF3, and 57.0% of neurons expressed GFP (Fig. 5g, h). Moreover, 98.2% of ATF3-expressing motor neurons also expressed GFP. In addition, examining the ventral root from L3 to S1, the most abundant levels of GFP-positive fibers were observed in the L6 root; the numbers of positive fibers became fewer in the roots next to L6 (Fig. 5i).

### The majority of GFP-expressing neurons in the RCS-loaded DRG are proprioceptors

**Fig. 6:**
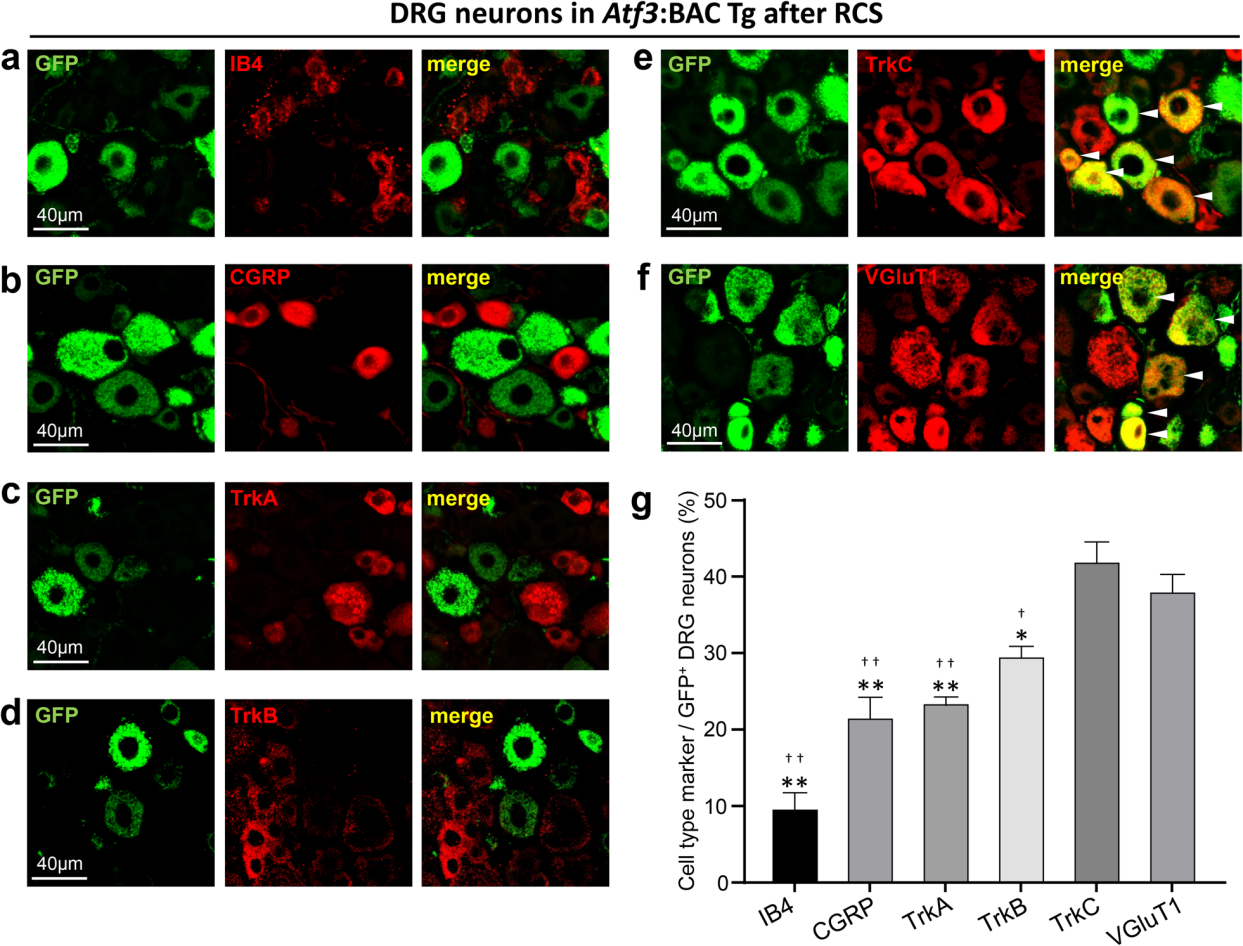
GFP is highly colocalized with proprioceptor markers in the DRG of *Atf3*:BAC Tg mouse after RCS. **a**–**f** Double immunostaining of GFP with various sensory neuron markers. IB4 is a marker of nociceptive neurons (**a**), CGRP is a marker of nociceptive neurons (**b**), TrkA is a marker of nociceptive neurons (**c**), TrkB is a marker of tactile/pressure sensation (**d**), TrkC is a marker of proprioceptor neurons (**e**), and VGluT1 is a marker of proprioceptor neurons (**f**). Note that co-localization can be strongly observed in **e** and **f** (arrowheads). **g** Co-expression rates of each marker with GFP-positive neurons in the DRG. *n* = 5 mice, *p < 0.001 vs. TrkC, **p < 0.0001 vs. TrkC, †*p* < 0.05 vs. VGluT1, ††*p* < 0.0001 vs. VGluT1. One-way ANOVA with Dunnett’s multiple comparisons test. Error bars denote the SEM. Scale bars, 40 μm

Using the *Atf3*:BAC Tg mouse, we next examined immunofluorescence staining with the following sensory neuron markers in conjunction with GFP: isolectin B4 (IB4), calcitonin gene-related peptide (CGRP), tropomyosin receptor kinase (Trk)A, TrkB, TrkC, and vesicular glutamate transporter 1 (VGluT1) after RCS (Fig. 6a–f). The marker that was most frequently co-expressed with GFP was TrkC, a marker of proprioceptive neurons, at a rate of 42.1%; this was followed by VGluT1, also a marker of proprioceptive neurons, at a rate of 38.2%. Although these two proprioceptive neuron markers had significantly higher percentages of co-localization with GFP, lower—but still significant—co-localization of other markers with GFP was also identified; namely, TrkB (29.7%) for tactile/pressure sensation, and TrkA, (23.6%), CGRP (21.7%), and IB4 (9.8%) for nociceptive neurons.

These results indicate that the majority of GFP-expressing cells in the DRG are proprioceptive neurons, and that fewer GFP-expressing cells are tactile and nociceptive neurons.

### GFP-positive fibers along the reflex arc of the spinal cord

The mitochondria of hyperactivated neurons were labeled with GFP. Given that mitochondria are located throughout the whole neuron, from axon tips to dendrite tips, the axon terminals and dendritic arborization of hyperactivated neurons were able to be visualized in the Tg mice.

**Fig. 7:**
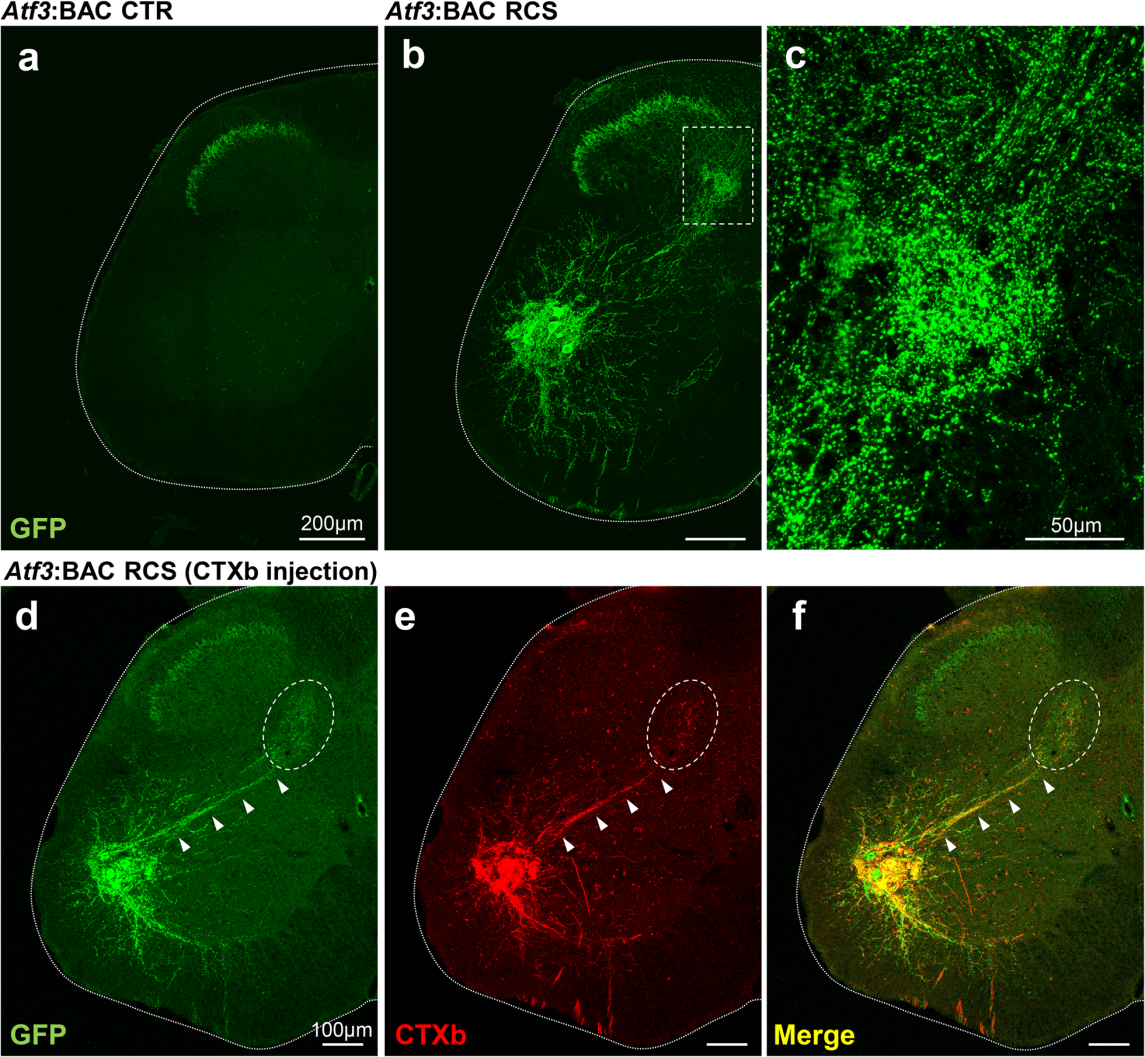
GFP-positive fiber trajectory in *Atf3*:BAC Tg mice after RCS exposure. **a**, **b** GFP expression in *Atf3*:BAC Tg mice before (**a**) and after (**b**) 7 days of RCS. Note that GFP expression can be observed from the medial part of the nucleus proprius (deep layers of the DH) to the motor neurons located in the dorsal part of the VH. The boxed area in **b** is magnified in **c**. **d–f** Injection of the neural tracer CTXb into the IMF of RCS-loaded *Atf3*:BAC Tg mice revealed the co-expression of GFP and CTXb in motor neurons and their fibers (arrowheads). GFP-positive fibers in the circled area may be central branch terminals from proprioceptive DRG neurons. The white dotted lines indicate the edge of spinal cord. Scale bars, 200 μm in **a, b**, 50 μm in **c**, 100 μm in **d–f**

After 7 days of RCS loading, GFP-positive fiber trajectories were clearly observed in the spinal cord. In the DH, GFP-positive fibers were present in the inner part of lamina II and the medial part of deeper laminae (Fig. 7a, b). The GFP-positive fibers observed in the inner part of lamina II may be central branch terminals from nociceptors and mechanoreceptors in the DRG, and the fibers in the medial part of deeper laminae may originate from proprioceptors in the DRG. The GFP-positive fibers in the inner part of lamina II were also observed in control mice. The expression of GFP did not change before and after RCS. The unexpected expression of GFP might result from the location of the transgene insertion. Therefore, we conclude that the expression of GFP in the inner part of lamina II is not involved in the alteration of PWT after RCS. In the VH, a cluster of motor neurons, with their wide dendritic arbors and axons, were positive for GFP. Notably, dense GFP-positive fibers were observed in the intermediate zone; they connected GFP-positive motor neurons with deeper laminae of the DH, indicating that GFP-positive processes were present along the reflex arc (Fig. 7c).

We next attempted to identify the target muscle of GFP-positive motor neurons as a final destination of the reflex arc. We injected a retrograde neuronal tracer, CTXb, into various hindlimb muscles of *Atf3*:BAC Tg mice after exposure to RCS. The CTXb injection into the IMF successfully led to the apparent double labeling of GFP and CTXb in motor neurons (Fig. 7d–f). These data suggest that motor neurons that express ATF3/GFP and are surrounded by microglia project to the IMF.

### GFP-positive neural fibers in muscle spindles and neuromuscular junctions of the IMF

Next, because GFP-positive fibers were observed along the spinal reflex arc in *Atf3*:BAC Tg mouse after RCS loading, we examined the existence of GFP fibers in muscle spindles and neuromuscular junctions of the IMF.

**Fig. 8:**
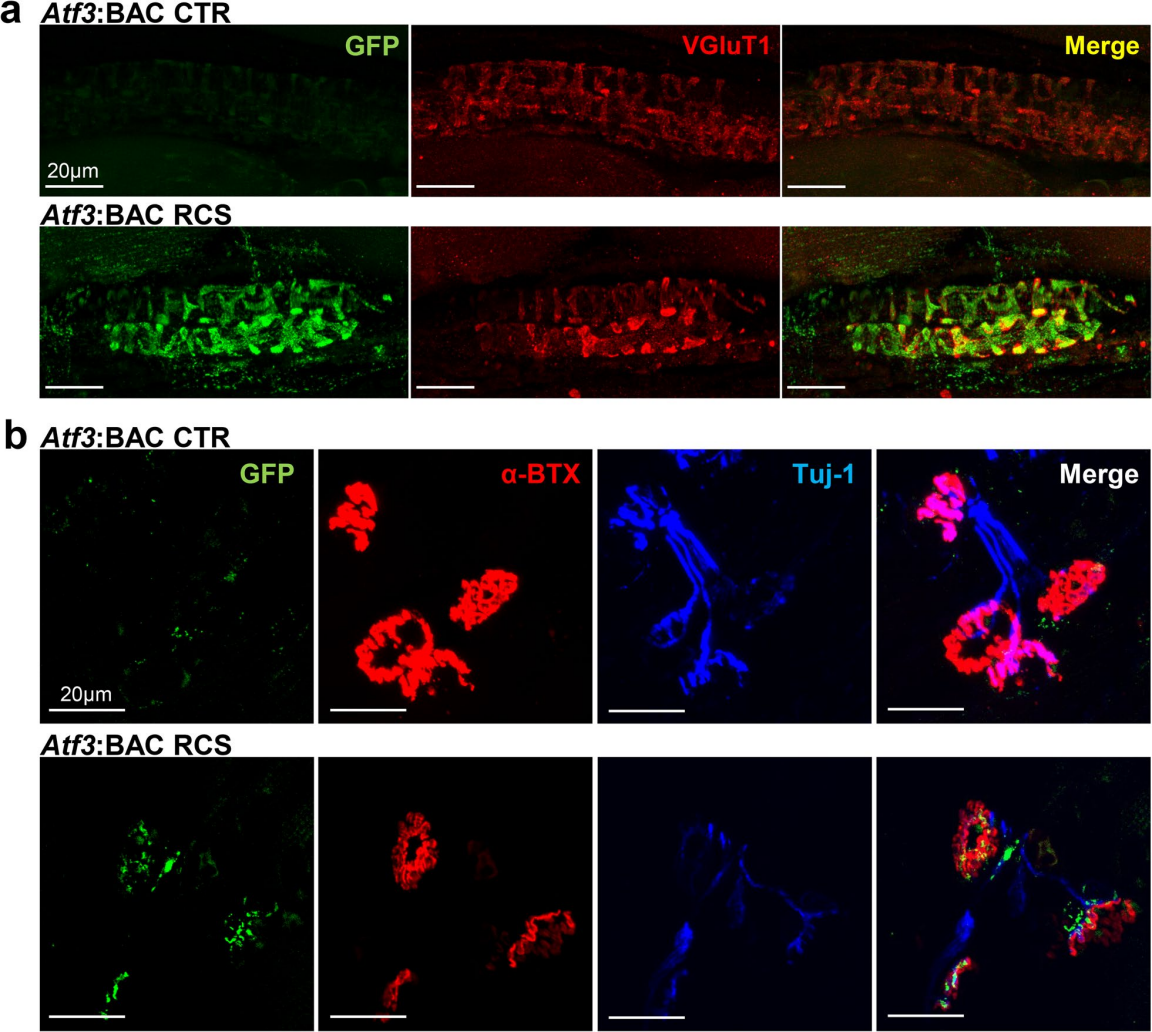
GFP-labeled fibers are observed along the nerve path of the reflex arc. **a** VGluT1-positive proprioceptive fibers (the peripheral branch of proprioceptor DRG neurons) in muscle spindles of the IMF were also positive for GFP in *Atf3*:BAC Tg mice after RCS. **b** GFP-positive fibers innervated the neuromuscular junctions in IMF of *Atf3*:BAC Tg mice after RCS. α-Bungarotoxin (α-BTX) labels ACh receptors, and class III beta-tubulin (Tuj1) shows axon terminals. Note that GFP-positive axons also innervated ACh receptors in the IMF. Scale bars, 20 μm

To identify muscle spindles, we used VGluT1, a marker of type Ia sensory fibers that perform proprioception. In *Atf3*:BAC Tg mouse, the VGluT1-positive sensory fibers in muscle spindles were also positive for GFP after RCS (Fig. 8a). Next, α-bungarotoxin, a marker of nicotinic acetylcholine (ACh) receptors at the neuromuscular junction, was used; we successfully confirmed that GFP-positive axons innervated ACh receptors in the IMF after RCS (Fig. 8b). We never observed GFP-positive muscle spindle nor GFP-positive neuromuscular junction in control condition of *Atf3*:BAC Tg mice.

### Microglial activation along the reflex arc

**Fig. 9:**
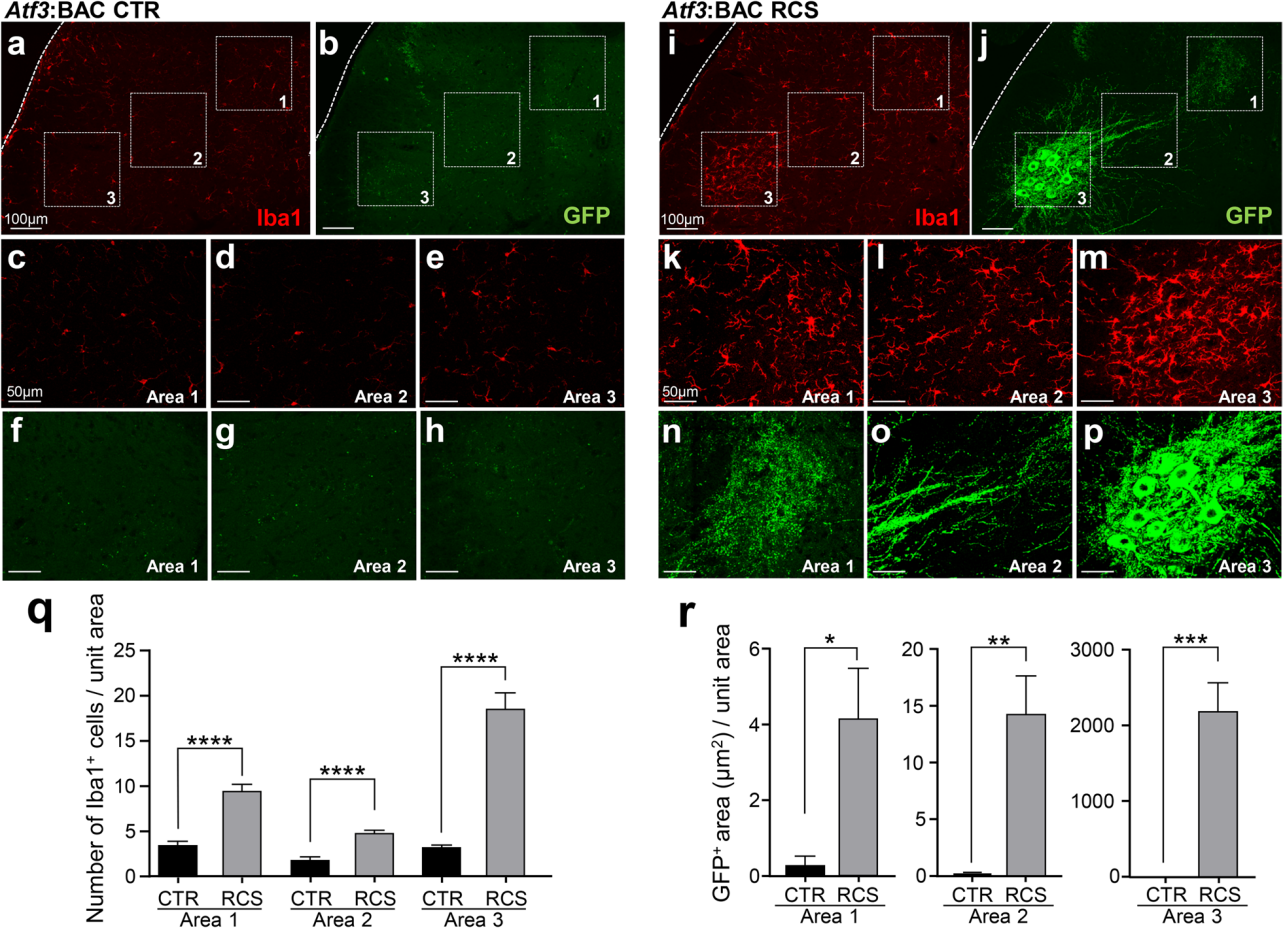
Microglia accumulate along the trajectory of the GFP-positive reflex arc in *Atf3*:BAC Tg mice. **a, b** Low-power magnification of Iba1 and GFP expression in the spinal cord of control (CTR) *Atf3*:BAC Tg mouse. Areas 1–3, surrounded by dotted boxes, indicate the areas for the images in **c–e** for Iba1 and **f–h** for GFP. **i, j** Low-power magnification of Iba1 and GFP expression in the spinal cord of RCS-loaded *Atf3*:BAC Tg mouse. Areas 1–3, surrounded by dotted boxes, indicate the areas for the images in **k–m** for Iba1 and **n–p** for GFP. **q** Quantification of the number of Iba1-positive cells in areas 1–3, shown in **a, i**. **r** GFP-positive area in areas 1–3, shown in **b, j**. *n* = 5 mice, *p < 0.05, **p < 0.01, ***p < 0.001, ****p < 0.0001. Error bars indicate the SEM. Unpaired t-test. Scale bars, 100 μm in **a, b, i, j** and 50 μm in **c–h, k–p**

In control spinal cord of *Atf3*:BAC Tg mouse, Iba1-positive microglia were sparsely distributed while GFP-positive signals were not observed (Fig. 9a–h). After RCS loading, microglial accumulation occurred along the trajectory of GFP-positive fibers (Fig. 9i–p). The microglial activation was observed in the medial part of deeper laminae of spinal cord (Fig. 9k). The GFP-positive fibers of RCS-loaded mice appeared in the corresponding area, in which proprioceptive neurons in the DRG terminate (Fig. 9n). The changes in microglial numbers and GFP expression were measured in three designated areas: the medial part of deeper laminae in the DH (area 1), the dorsal VH where GFP-positive motor neurons accumulated (area 3), and the region connecting these two regions (intermediate column, area 2) (Fig. 9a, b, i, j). In the Tg mice, both the GFP expression area and the microglial number were significantly increased in all areas compared with the control group (Fig. 9q, r).

### Suppression of microglial activation reduces pain in the RCS model

**Fig. 10:**
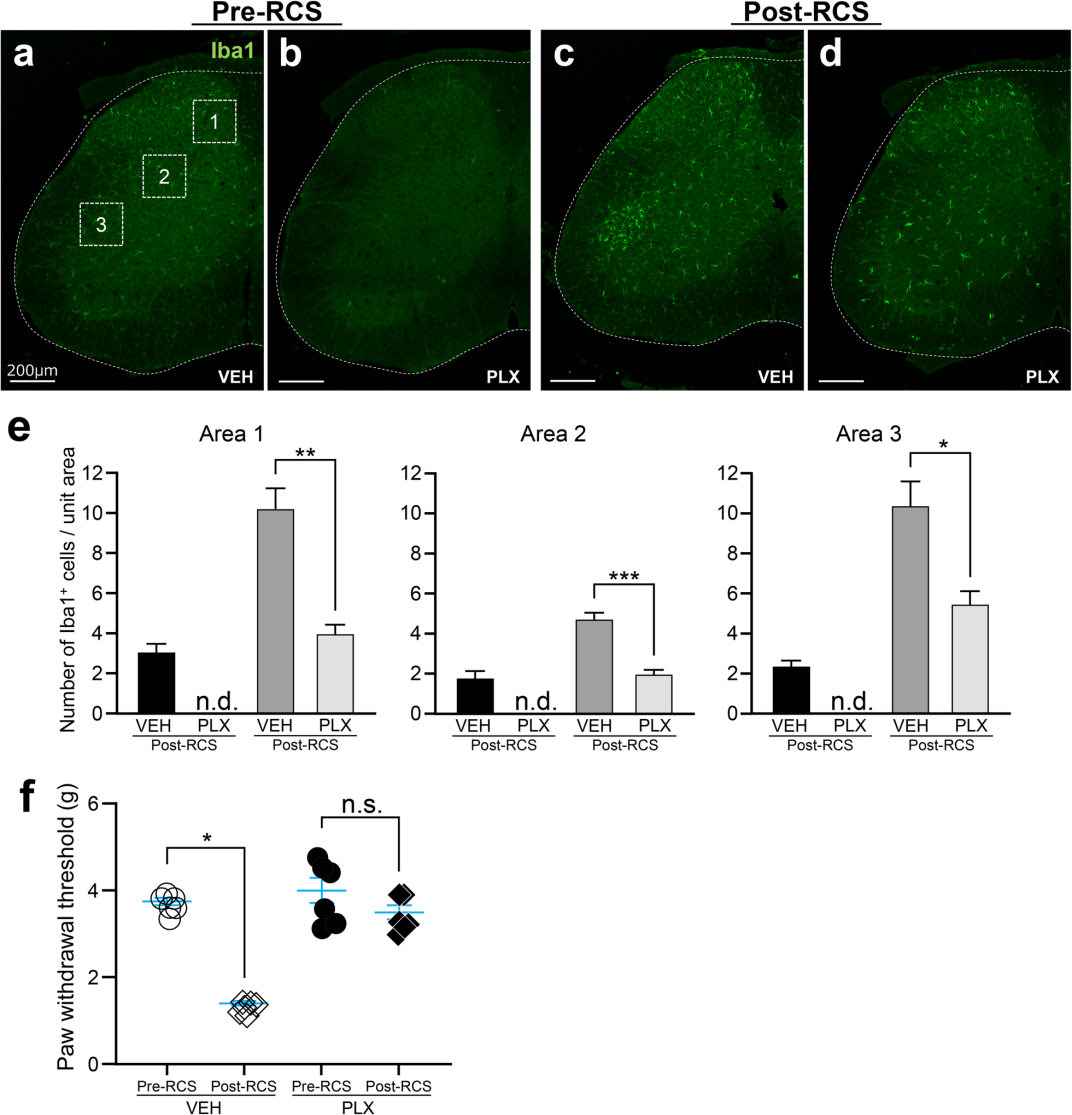
Elimination of microglia by PLX3397 improves RCS-induced pain behavior. **a** A vehicle-treated mouse (VEH), showing the normal localization of Iba1-positive microglia. **b** Mice treated with 3 days of PLX3397 (PLX) before RCS (Pre-RCS), showing that almost all microglia have disappeared. The areas corresponding to the medial part of the nucleus proprius in the DH (area 1), the intermediate column (area 2), and the dorsal part of the VH (area 3) were used for the quantification in **e**. **c** After 7 days of RCS loading (Post-RCS) with vehicle treatment (VEH), there was the microglial accumulation in both the DH and VH. **d** Similar accumulations of Iba1-positive microglia were not observed after RCS with PLX treatment, although a small number of activated Iba1-positive microglia were spread evenly throughout the spinal cord.** e** Numbers of Iba1-positive cells in areas 1–3, shown in **a**. *n* = 5 mice for pre-RCS VEH and PLX3397 group. *n* = 6 mice for post-RCS VEH and PLX3397 group. *p < 0.01, **p < 0.001, ***p < 0.0001. Error bars indicate the SEM. Unpaired *t*-test. **f** Changes in paw withdrawal with microglial elimination in the PLX3397 group. *n* = 6 mice, *p < 0.0001. Error bars indicate the SEM, n.d., not detected and n.s., not significant. Paired *t*-test. The white dotted lines indicate the edge of spinal cord in **a–d**. Scale bars = 200 μm in **a–d**

To suppress RCS-induced microglial activation, PLX3397—a known inhibitor of colony-stimulating factor-1 receptor—was used. PLX3397 administration for 3 days completely removed microglia from the mouse spinal cord (Fig. 10a, b). After 3 days of oral PLX3397 administration under normal conditions, mice underwent RCS. During RCS, PLX3397 was continuously administered every 12 h. Microglial accumulation in the spinal cord was significantly reduced in the PLX3397-treated group compared with the vehicle-treated group after RCS (Fig. 10c, d), although a few activated microglia were observed even after PLX3397 treatment under RCS. Microglial proliferation was significantly suppressed by PLX3397 treatment in all 1–3 areas (Fig. 10e).

Concomitant with the suppression of microglial accumulation, PLX3397 treatment led to an increased mechanical withdrawal threshold after RCS, indicating that microglial suppression reduces pain behavior in the RCS model (Fig. 10f).

## Discussion

The present study demonstrated a possible mechanism underlying chronic pain in a fibromyalgia model mouse. The RCS-treated mice demonstrated that proprioceptor hyperactivation leads to local microglial activation along the spinal reflex arc, and that prolonged microglial activation may be responsible for chronic pain (Additional file 4: Fig. S3). Similar proprioceptor-induced chronic pain has also been demonstrated in an ME/CFS model mouse, where the different stress-loading paradigm also activated microglia in a proprioceptor-mediated manner. It is intriguing that proprioceptor-induced microglial activation may be the common cause of chronic pain in both fibromyalgia and ME/CFS models, although the methods used were somewhat different.

The relevance of the RCS model as a fibromyalgia model has been addressed by several papers using rats and mice [14–17]. All of these animal models showed bilateral hindlimb allodynia; furthermore, systemic gabapentin treatment had complete anti-allodynic effects in this model [17]. Notably, Montserrat-de la Paz et al. [16] also demonstrated behavioral changes related to cognitive disturbances, anxiety, and depression. Together, these findings support the relevance of the RCS model as a fibromyalgia model.

In the present study, we used the recently established *Atf3:*BAC Tg mouse, in which almost all mitochondria are labeled by GFP in conjunction with ATF3 expression when neurons are hyperactivated or injured [18]. The ATF3 expression is specifically induced in response to neuronal injury [19, 25, 26], although hyperactivation also induces ATF3 expression. Although cFos is often used as a stress or activation marker of neurons, it is so sensitive that slight stimuli can also induce its expression. By contrast, the present study demonstrated that ATF3 expression only occurs in relatively severe conditions, such as those of hyperactivation and axon injury. ATF3 expression has also been reported in amyotrophic lateral sclerosis model mice; motor neurons express ATF3 in the symptomatic stage [27]. Using the advantages of ATF3 expression, the *Atf3*:BAC Tg mouse was designed to identify hyperactivated and injured neurons. Using this Tg mouse, it was possible to clearly observe the trajectory of the hyperactivated neuronal circuit because GFP-labeled mitochondria were present throughout whole neurons, from the tips of dendrites to the tips of axons. The present findings demonstrated the existence of GFP-positive signals in muscle spindles of the IMF, predominantly in proprioceptors among DRG neurons, motor neurons projecting to the IMF, and neuromuscular junctions of the IMF. Together, these results suggest that RCS can activate the neuronal circuit of the spinal reflex arc.

The origin of the proprioceptive stimulus in the current model was the foot muscle, rather than the leg and/or thigh. In our previous study using a CFS/ME model, the soleus was used as the origin. Because the paw directly touches the cold floor, we initially thought that a cold temperature to the paw might cause inflammation or injury; however, we did not observe any induction of IL-6, IL-1β, or TNF-α mRNAs—which are associated with inflammation—in the paw skin or the IMF. Furthermore, neither systemic inflammatory signatures such as C-reactive protein nor muscle damage markers such as creatine kinase levels were increased in the peripheral blood with RCS (Additional file 3: Fig. S2). In addition, it has been reported that the intramuscular application of neurotrophic factors, such as nerve growth factor and glial cell-derived neurotrophic factor, induce a mechanical response of nociceptors in muscles [28, 29]; however, the mRNAs for nerve growth factor, glial cell-derived neurotrophic factor, brain-derived neurotrophic factor, artemin, neurturin, persephin, and neurotrophin-3 are reportedly unchanged in the muscles of an RCS model [30]. This finding suggests that a non-inflammatory, non-nociceptive, but hypertonic condition may occur in the muscle, although we were unable to explore why the IMF might be the target. We hypothesize that the repetitive tonus and relaxation of the muscle may be implicated in eliciting proprioceptor hyperactivation because constant (rather than intermittent) cold stress does not lead to chronic pain [17].

Although the experimental paradigms in the fibromyalgia and CFS/ME models are completely different (the present study and Yasui et al. [13]), the events that occur in the spinal cord appear very similar [31]; both spinal reflex arc activation and subsequent microglial activation along the reflex arc occur. The involvement of microglia in pain seems clear in both models, and microglial inhibition succeeded in reducing pain behavior in both of the models. The mechanisms revealed in these two models suggest that unconscious, repetitive, and chronic stimuli to a specific muscle can cause continuous proprioceptor hyperactivation, and that subsequent microglial activation may lead to chronic pain. We cannot exclude the possibility that depression and anxiety are also involved in pain signal procession in RCS model [32, 33]. Detailed mechanism will be investigated in further study.

## Conclusions

The present study revealed that proprioceptor-induced microglial activation is the underlying mechanism of chronic pain in the fibromyalgia model. Intriguingly, the mechanism is the same as that of a CFS/ME model despite differences in the experimental paradigms. The mechanisms underlying chronic pain in other FSSs may be extrapolated from the present data: namely, proprioceptor-induced microglial activation. Other or additional possibilities for the mechanisms of chronic pain remain; for example, the involvement of the limbic system and frontal cortex is likely. Nevertheless, the present mechanism demonstrated at the spinal cord level may be critical, and is a potential therapeutic target.

### Supplementary Information

**Additional file 1: Table S1.** Antibody information.

**Additional file 2: Figure S1. (a)** The PWT of the female RCS group was lower than that of the untreated control (CTR) group on day 5 of RCS loading. *n* = 3 mice for each group. **(b)** A significant accumulation of microglia (Iba1-positive cells) and the expression of ATF3 were induced in the spinal cord of female mouse on day 7 of RCS loading. The white dotted lines indicate the edge of spinal cord. Scale bars = 200 μm.

**Additional file 3: Figure S2. (a)** The amount of C-reactive protein (CRP) and creatine kinase (CK), typical markers of inflammation, in peripheral blood samples from control and RCS mice. *n* = 6 mice. **(b)** mRNA expression of IL-1β, IL-6, and TNF-α in the hind paw skin of control and RCS mice. **(c)** mRNA expression of IL-1β, IL-6, and TNF-α in the IMF of control and RCS mice. *n* = 3 mice. 3 independent experiments were performed. Error bars indicate the SEM.

**Additional file 4: Figure S3.** Schematic diagram of the pathway from the spinal cord to the peripheral nerves of the muscle in RCS.

## Data Availability

The datasets used and/or analyzed during the current study are available from the corresponding author on reasonable request.

## Abbreviations

RCS: Repeated cold stress
BAC: Bacterial artificial chromosome
ATF: Activating transcription factor
DRG: Dorsal root ganglion
IMF: Intrinsic muscles of the foot
CFS: Chronic fatigue syndrome
ME: Myalgic encephalomyelitis
FSS: Functional somatic syndrome
ICS: Intermittent cold stress
Tg: Transgenic
GFP: Green fluorescent protein
PWT: Paw withdrawal threshold
CTXb: Cholera toxin B subunit
α-BTX: α-Bungarotoxin
