# Supplementary figures and images for: Repeated cold stress, an animal model for fibromyalgia, elicits proprioceptor-induced chronic pain with microglial activation in mice
Source: J Neuroinflammation. 2024 Jan 18;21:25. doi: 10.1186/s12974-024-03018-6 (PMC10795366; doi:10.1186/s12974-024-03018-6)

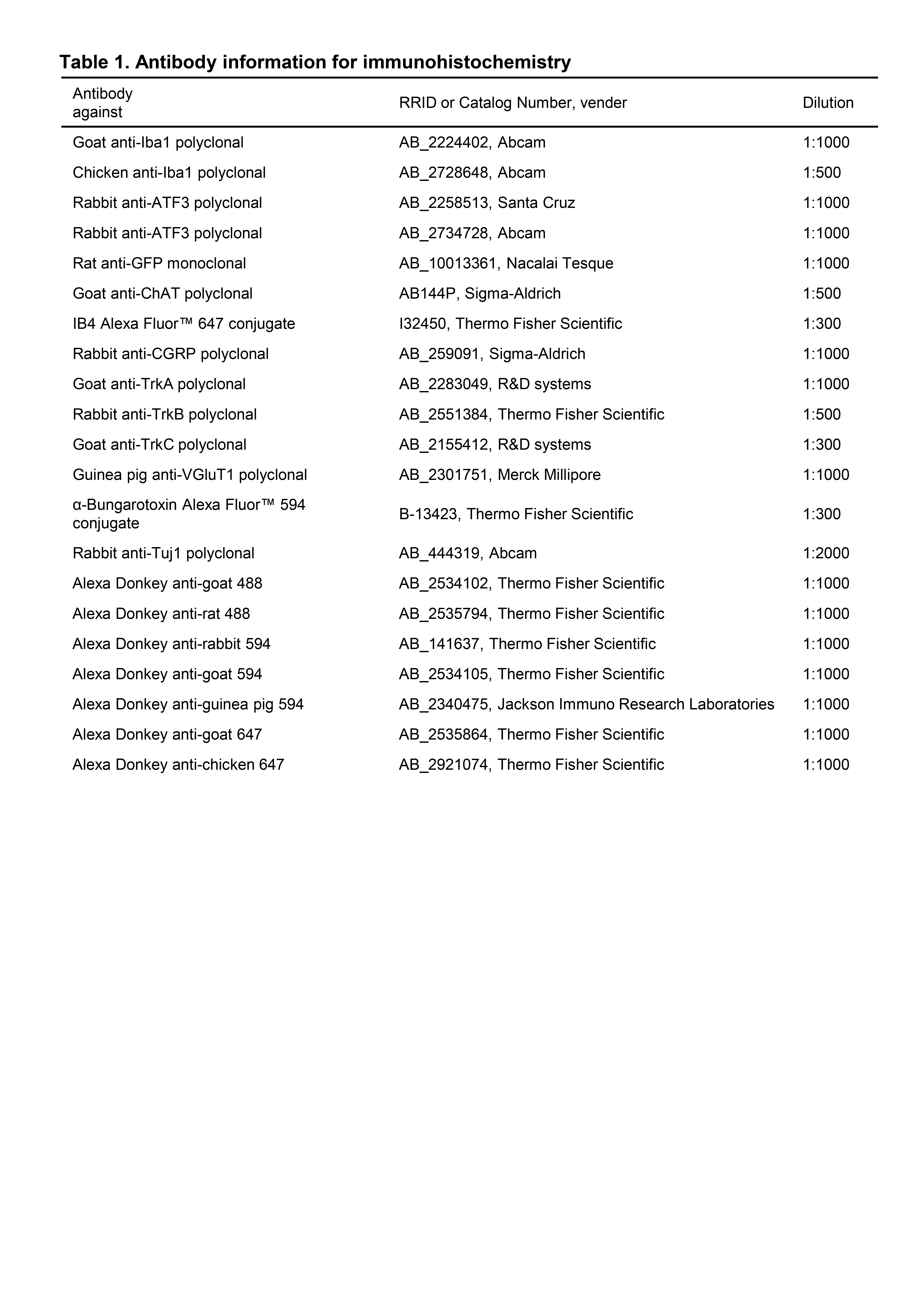

Supplement: Supplementary file 1 — Additional file 1: Table S1. Antibody information. [file 12974_2024_3018_MOESM1_ESM.tif]

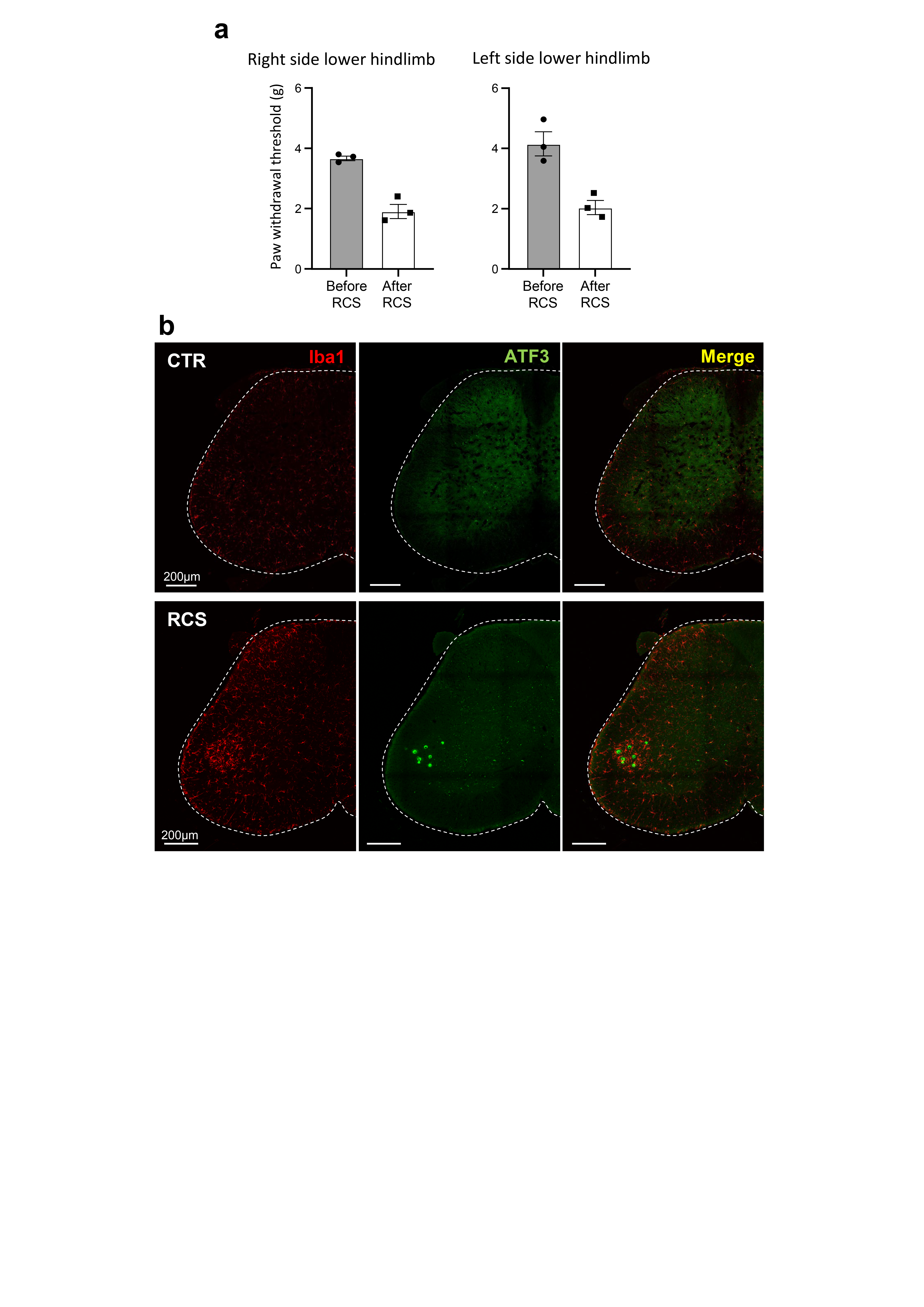

Supplement: Supplementary file 2 — Additional file 2: Figure S1. (a) The PWT of the female RCS group was lower than that of the untreated control (CTR) group on day 5 of RCS loading. n = 3 mice for each group. (b) A significant accumulation of microglia (Iba1-positive cells) and the expression of ATF3 were induced in the spinal cord of female mouse on day 7 of RCS loading. The white dotted lines indicate the edge of spinal cord. Scale bars = 200 μm. [file 12974_2024_3018_MOESM2_ESM.tif]

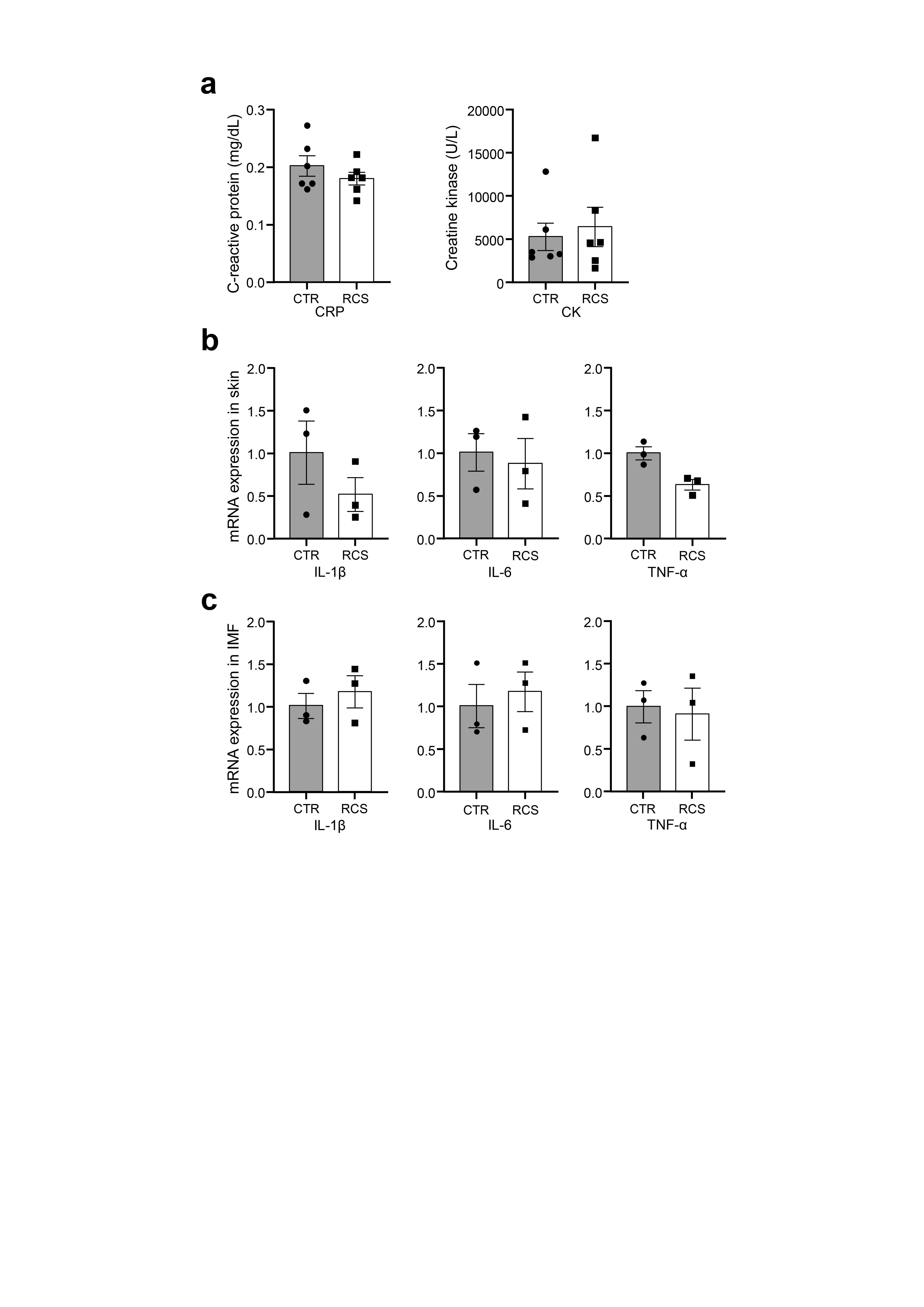

Supplement: Supplementary file 3 — Additional file 3: Figure S2. (a) The amount of C-reactive protein (CRP) and creatine kinase (CK), typical markers of inflammation, in peripheral blood samples from control and RCS mice. n = 6 mice. (b) mRNA expression of IL-1β, IL-6, and TNF-α in the hind paw skin of control and RCS mice. (c) mRNA expression of IL-1β, IL-6, and TNF-α in the IMF of control and RCS mice. n = 3 mice. 3 independent experiments were performed. Error bars indicate the SEM. [file 12974_2024_3018_MOESM3_ESM.tif]

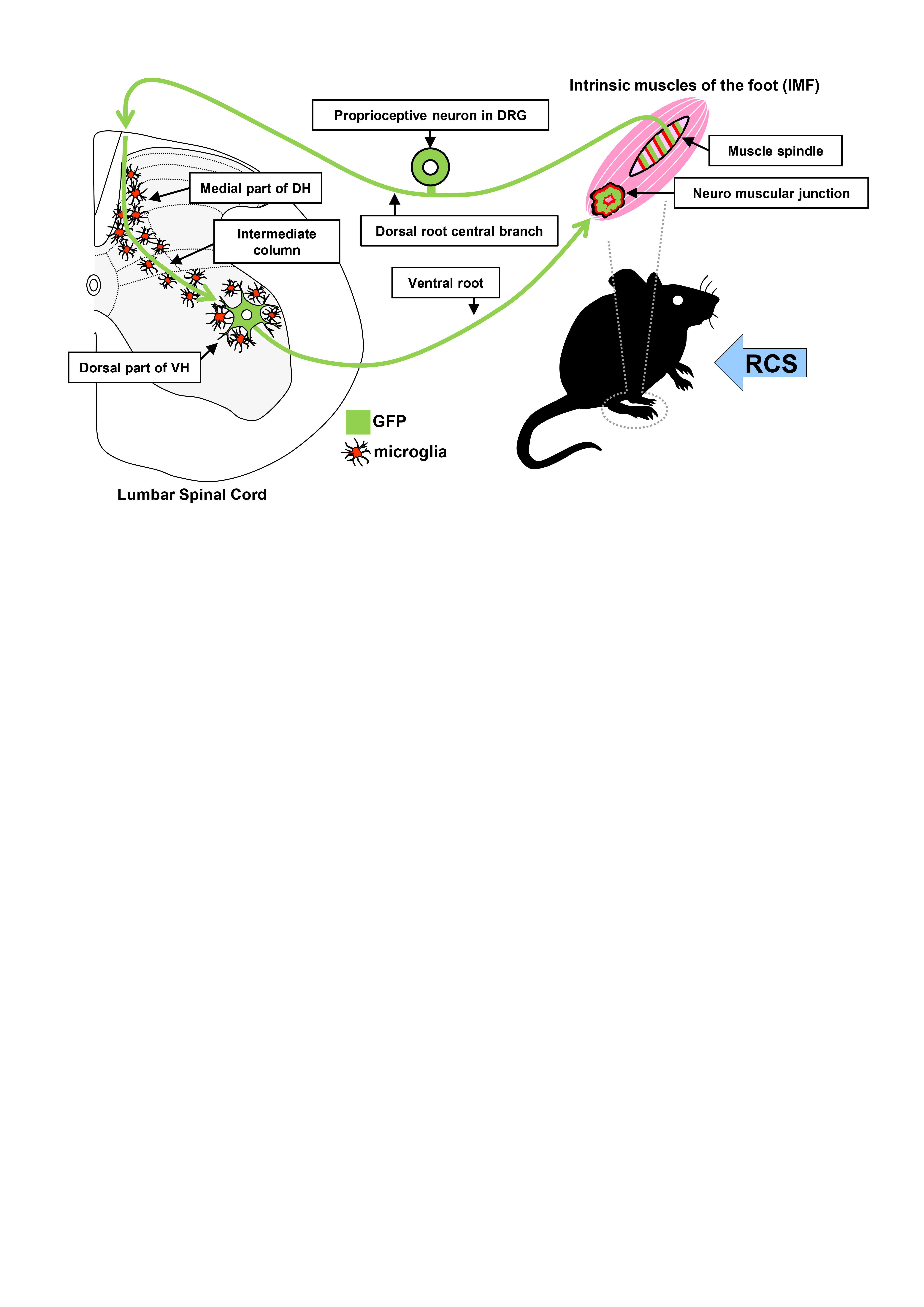

Supplement: Supplementary file 4 — Additional file 4: Figure S3. Schematic diagram of the pathway from the spinal cord to the peripheral nerves of the muscle in RCS. [file 12974_2024_3018_MOESM4_ESM.tif]
